# Die informierte Entscheidung als Ziel von evidenzbasierten Gesundheitsinformationen: Das Beispiel Krebsfrüherkennung

**DOI:** 10.1007/s00103-022-03526-x

**Published:** 2022-04-26

**Authors:** Milly Schröer-Günther, Klaus Koch

**Affiliations:** Institut für Qualität und Wirtschaftlichkeit im Gesundheitswesen (IQWiG), Im Mediapark 8, 50670 Köln, Deutschland

**Keywords:** Reihenuntersuchung, Gesundheitsinformation, Entscheidungshilfe, Informierte Endscheidung, Evidenzbasiert, Mass screening, Consumer health information, Decision aid, Informed decision, Evidence-based

## Abstract

Um Menschen eine informierte Entscheidung über die Teilnahme an Krebsfrüherkennungsuntersuchungen zu ermöglichen, müssen neben den Vorteilen auch Nachteile wie medizinische Risiken kommuniziert werden. Die in solchen Entscheidungshilfen enthaltenen Informationen sollten evidenzbasiert, neutral, ausgewogen und verständlich sowie in ihrem Umfang angemessen sein. Das Institut für Qualität und Wirtschaftlichkeit im Gesundheitswesen (IQWiG) wurde in den Jahren 2014 und 2015 vom Gemeinsamen Bundesausschuss (G-BA) beauftragt, Entscheidungshilfen in Form von Broschüren zur Teilnahme am Mammografie‑, Darmkrebs- und Zervixkarzinomscreening zu erstellen, die inzwischen im Einsatz sind.

In diesem Beitrag wird die Entwicklung der Entscheidungshilfen beschrieben, wobei der Fokus auf deren Inhalten und den Ergebnissen der extern durchgeführten Nutzertestungen liegt. Letztere ergaben, dass 10–20 % der Testerinnen und Tester nach dem Lesen ihre Einstellung zur Teilnahme an der Früherkennungsuntersuchung innerhalb der 3 Kategorien „teilnahmebereit“, „unentschlossen“ und „nicht teilnahmebereit“ geändert haben. Das weist darauf hin, dass eine informierte Entscheidung durch die Materialien unterstützt wird. Die Nutzertestungen trugen dazu bei, die Entscheidungshilfen noch besser an die Informationsbedürfnisse der angesprochenen Gruppen anzupassen.

## Abstract (en)

In order to enable people to make an informed decision about participating in cancer screening, disadvantages like medical risks have be communicated in addition to advantages. The information in the decision aids should be evidence-based, neutral, balanced, understandable, and adequate in scope. The Institute for Quality and Efficiency in Health Care (IQWiG) was commissioned by the Federal Joint Committee (G-BA) in 2014 and 2015 to develop decision aids in the form of brochures on participation in mammography, colorectal cancer, and cervical cancer screening, which are now in use.

This article describes the development of the decision aids, with a focus on the results of user tests, which were executed by an external provider. Of the testers, 10 to 20% changed their attitude toward taking part in the screening test within the three categories “willing to take part,” “undecided,” and “not willing to take part” after reading the brochures. This indicates that an informed decision is supported by the materials. The user tests helped to better adapt the decision aids to the information needs of target groups.

## Einleitung

Die Website gesundheitsinformation.de ist ein Angebot des Instituts für Qualität und Wirtschaftlichkeit im Gesundheitswesen (IQWiG). Das IQWiG hat den gesetzlichen Auftrag, für erkrankte sowie für gesunde Bürgerinnen und Bürger kostenlos unabhängige und wissenschaftlich geprüfte Informationen zur Verfügung zu stellen. Dieser Auftrag wird vor allem über 2 Wege umgesetzt.

Zum einen erarbeitet und aktualisiert das IQWiG Informationen für einen Katalog von Themen, der insbesondere häufige Krankheiten, Diagnosen und Gesundheitsfragen umfasst. Dieser wird den Bürgerinnen und Bürgern auf der Website gesundheitsinformation.de zur Verfügung gestellt. Die Website umfasst derzeit Informationspakete zu gut 200 Erkrankungen und Behandlungsmöglichkeiten. Das IQWiG erhält zum Zweiten auch direkt vom Gemeinsamen Bundesausschuss (G-BA) oder vom Bundesministerium für Gesundheit (BMG) Aufträge, Patienteninformationen zu bestimmten Themen zu erstellen.

Methoden und Prozesse der Erstellung leiten sich generell aus der Verpflichtung des IQWiG gegenüber der evidenzbasierten Medizin ab. Für Gesundheitsinformationen bedeutet das, dass zum einen die Inhalte der Informationen dem aktuellen Stand des Wissens entsprechen müssen. Zum anderen folgen die Gestaltung und Kommunikation konsentierten Qualitätsanforderungen. Ziele der Gesundheitsinformationen des IQWiG sind u. a., das Verständnis von Vor- und Nachteilen wichtiger Behandlungsmöglichkeiten zu erhöhen und die Gesundheitskompetenz zu stärken.

Das IQWiG wurde im Jahr 2014 vom G‑BA mit der Erstellung eines Einladungsschreibens und einer Entscheidungshilfe zum Mammografiescreening beauftragt (Projektnummer P14-03; [1]). Ein Jahr später folgten Aufträge zum Darmkrebsscreening (Projektnummer P15-01; [2]) und zum organisierten Zervixkarzinomscreening (Projektnummer P15-02; [3]).

Diese Früherkennungsprogramme sind im § 25 des Fünften Sozialgesetzbuches (SGB V) verankert. Dieser Paragraf wurde 2013 mit dem „Krebsfrüherkennungs- und -registergesetz – KFRG“ reformiert [4] und dabei die gesetzliche Anforderung verankert, dass eine „umfassende und verständliche Information der Versicherten über Nutzen und Risiken der jeweiligen Untersuchung“ erfolgen muss [5]. In der Gesetzesbegründung dieser Änderung formulierte der Gesetzgeber klare Ansprüche an die Inhalte der Information:

Denn auch bevölkerungsmedizinisch sinnvolle und empfehlenswerte Krebsfrüherkennungsmaßnahmen beinhalten für die gesunde bzw. beschwerdefreie Person ein Risiko. Hierzu gehören – neben den Risiken der Untersuchung selbst – die Konsequenzen falsch-negativer oder falsch-positiver Testbefunde, invasive Abklärungsuntersuchungen (z. B. die Entnahme von Gewebeproben) sowie die mögliche Diagnose und Behandlung von Krebserkrankungen, von denen die Person ohne die Früherkennung in ihrem Leben nie etwas gemerkt hätte. Solche Risiken lassen sich auch durch die bestmögliche Qualitätssicherung nicht in allen Fällen vermeiden, sondern allenfalls minimieren. Daher sollte das Inanspruchnahmeverhalten der einzelnen Person allein durch eine ausreichende, neutrale und verständliche Information und Beratung sowie deren individuelle Werte und Präferenzen bestimmt sein und nicht durch Anreizsysteme beeinflusst werden. Dies entspricht auch den Empfehlungen des Rates der Europäischen Union, der Europäischen Leitlinien und der Expertinnen und Experten des Nationalen Krebsplans, welche die eigenständige, freiwillige und informierte Entscheidung über die Teilnahme in den Vordergrund stellen. Hierbei ist das Ziel einer informierten individuellen Entscheidung dem Ziel einer möglichst hohen Teilnahmerate übergeordnet [6].

Tatsächlich entspricht diese Neutralität auch dem Kernverständnis evidenzbasierter Gesundheitsinformationen, die Patientinnen und Patienten unterstützen wollen, informierte gesundheitliche Entscheidungen zu treffen [7, S. 135 ff.]. Die Mehrzahl der Patientinnen und Patienten bevorzugt es, solche Entscheidungen gemeinsam mit Ärztinnen, Ärzten und anderen Gesundheitsfachkräften vorzubereiten. Diese sogenannte gemeinsame Entscheidungsfindung kann durch Entscheidungshilfen (Decision Aids) unterstützt werden. Dabei handelt es sich um spezielle Informationsformate, wie z. B. Broschüren oder Fragebögen, die Nutzerinnen und Nutzer befähigen sollen, gemeinsam mit Ärztinnen und Ärzten oder Angehörigen anderer medizinischer Berufsgruppen informierte, den persönlichen Präferenzen entsprechende medizinische Entscheidungen zu treffen [1, 8]. Studien zeigen, dass Entscheidungshilfen Wissen vermehren und die Risikoeinschätzungen verbessern, Entscheidungskonflikte mindern und die Einbindung im Sinne einer gemeinsamen Entscheidungsfindung (Shared Decision Making – SDM) fördern können [1, 8]. Auch Entscheidungshilfen zur Krebsfrüherkennung können sich positiv auf die Entscheidungsfindung auswirken [9]. Menschen, die Entscheidungshilfen nutzen, sind zufriedener mit den erlebten Entscheidungsprozessen.

Die vorliegende Arbeit beschreibt den Entwicklungsprozess der 3 Entscheidungshilfen zu den Krebsfrüherkennungsprogrammen. Dabei werden ausgewählte Ergebnisse der qualitativen und quantitativen Nutzertestungen präsentiert und diskutiert.

## Entwicklung der Entscheidungshilfen zu Krebsfrüherkennungsuntersuchungen

Die Erstellung der Entscheidungshilfen folgte den Methoden und Prozessen des IQWiG zur Erstellung von Gesundheitsinformationen [7], den Anforderungen der International Patient Decision Aid Standards (IPDAS; [10]), der Guten Praxis Gesundheitsinformation [11] sowie den Best-Practice-Beispielen zur Erstellung von Entscheidungshilfen [12, 13]. Bei der Umsetzung der Aufträge wurden vom IQWiG klinische und methodische Sachverständige einbezogen. Die grundlegende Methodik war in allen 3 Projekten identisch, sodass sich folgende Arbeitsschritte ergaben:

1. eine systematische Literaturrecherche nach qualitativen Studien zur Ermittlung der Informationsbedürfnisse der Zielgruppe,
2. eine systematische Literaturrecherche zur Bewertung der Evidenz auf Basis von systematischen Übersichten oder ggf. Modellierung für Langzeiteffekte (P15-02 Zervixkarzinom),
3. Entwicklung der Entwürfe für Einladungsschreiben und Entscheidungshilfen,
4. qualitative Nutzertestung der erstellten Materialien inklusive Experteninterviews mit Ärztinnen und Ärzten sowie medizinischen Fachangestellten,
5. Einarbeitung der Ergebnisse aus qualitativen Nutzertestungen,
6. öffentliches Stellungnahmeverfahren,
7. quantitative Nutzertestungen der präfinalen Materialien und Projektabschluss.

Die qualitativen Nutzertestungen sowie die quantitativen Nutzertestungen wurden von einem externen Anbieter durchgeführt. Die Teilnehmerinnen und Teilnehmer der quantitativen Nutzertestungen wurden in Abhängigkeit von den Altersgrenzen des Screeningprogramms nach Alter quotiert. Personen mit niedriger Bildung (höchstens Hauptschulabschluss) wurden überquotiert. Die Methoden sind ausführlich in den online verfügbaren Abschlussberichten der einzelnen Projekte beschrieben [1–3].

## Ergebnisse der qualitativen und quantitativen Nutzertestungen

In dieser Arbeit werden ausgewählte Ergebnisse der qualitativen und quantitativen Nutzertestungen präsentiert und diskutiert. Die vollständigen Ergebnisse sind in den Abschlussberichten der Projekte dargestellt [1–3].

### Entscheidungshilfe zum Mammografiescreening

Im Rahmen des Mammografiescreeningprojektes wurde das seit 2010 im Mammografieprogramm eingesetzte Merkblatt zu einer Entscheidungshilfe für Frauen im Alter von 50 bis 69 Jahren weiterentwickelt. Schwerpunkte der Weiterentwicklung waren:

- Darstellung der Vor- und Nachteile,
- Beschreibung von Überdiagnosen,
- tabellarische und grafische Veranschaulichung der Vor- und Nachteile,
- Erstellung eines Instruments zur Unterstützung der Präferenzklärung (eine Art Fragebogen zur Reflexion verschiedener Optionen und der eigenen Präferenz).

Das Präferenzklärungsinstrument hat das Ziel, den Frauen die Möglichkeit zu geben, über die mit der Entscheidung zusammenhängenden Aspekte (z. B. Wahrscheinlichkeit einer Fehldiagnose) nachzudenken und die bevorzugte Option (z. B. Teilnahme am Screeningprogramm – Ja oder Nein?) zu identifizieren [14, 15].

#### Qualitative Nutzertestung.

Insgesamt wurde die Entscheidungshilfe in der qualitativen Nutzertestung mit 4 Fokusgruppen (*n* = 37 Frauen) im Wesentlichen (31 von 37 Personen) als gut verständlich und hilfreich für die Entscheidungsfindung beurteilt. Das Präferenzklärungsinstrument bewerteten 18 der 37 Frauen als hilfreich. 15 Frauen hingegen bewerteten das Präferenzklärungsinstrument als nicht hilfreich. Die Begründung lag darin, dass die befragten Frauen bereits vorab eine Entscheidung getroffen beziehungsweise starke Präferenzen und deshalb keinen Bedarf an dieser Unterstützung hatten. Es wurden keine Themen genannt, die in dem Präferenzklärungsinstrument als überflüssig wahrgenommen wurden.

Als schwierig erwies sich die Vermittlung der Bedeutung von Überdiagnosen. Nur wenigen befragten Frauen waren „Überdiagnosen“ und ihre Folgen bekannt. Die Information über die Existenz von Überdiagnosen wurde von fast allen Teilnehmerinnen einerseits als beunruhigend, andererseits aber auch als relevant und wichtig empfunden. Zur besseren Vermittlung wurde die Anregung der Testerinnen aufgenommen, Überdiagnosen anhand einer Grafik zu erklären. Dabei wurde auf Vorarbeiten der australischen Entscheidungshilfe „Breast cancer screening: It’s your choice“ zurückgegriffen [16].

#### Quantitative Nutzertestung.

Im Rahmen der quantitativen Nutzertestung wurden Frauen zweier Altersgruppen befragt (Frauen < 50 Jahre und Frauen zwischen 50 und 69 Jahren). Zusätzlich zu den Frauen, die die Einladung zum Screening erhalten (> 50 Jahre), wurde entschieden auch Frauen zu befragen, die noch nicht mit der Entscheidung bzw. der Einladung konfrontiert waren, für die beides aber bald ansteht. Die Entscheidungshilfe wurde von den meisten befragten Frauen (ca. 80 %) als sehr gut bewertet. Die Inhalte wurden zumeist als sehr vertrauenswürdig empfunden (über 80 %).

**Figure 1:**
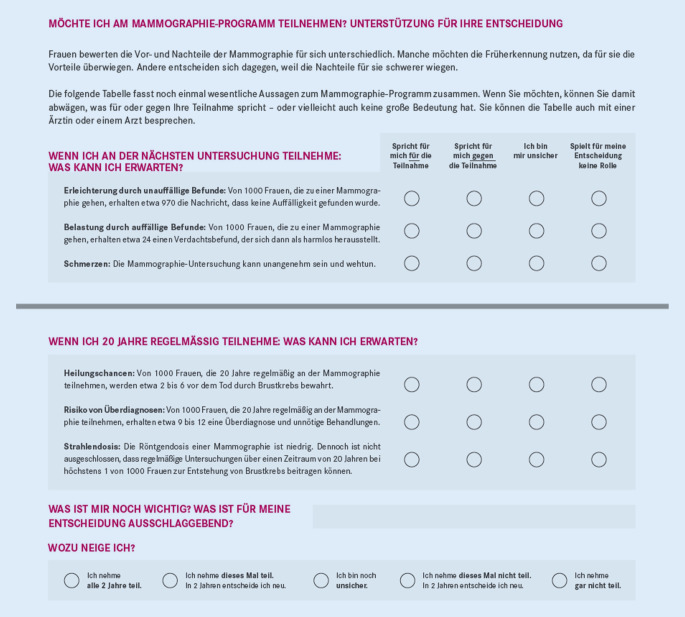

Das *Wissen zu Brustkrebs *wurde vor und nach dem Lesen der Broschüre abgefragt. Nach dem Lesen der Broschüre gingen deutlich mehr Frauen von einer geringeren Brustkrebshäufigkeit aus als noch davor (Zuwachs bis zu 15 %). Die Broschüre senkt damit die Risikowahrnehmung. Durch die Wissensfragen zeigte sich darüber hinaus, dass mehr Frauen nach dem Lesen der Broschüre die Frage, was mit Überdiagnosen gemeint ist, richtig beantworteten (vorher 12 % versus nachher rund 60 %). Das Präferenzinstrument wurde von vielen – besonders von im Vorfeld unentschlossenen – Frauen als hilfreich bewertet (Abb. 1).

**Figure 2:**
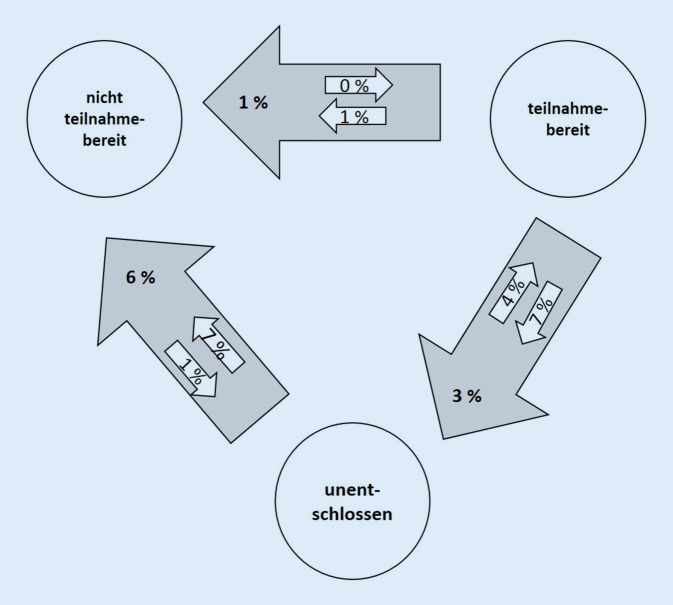

Die *Teilnahmebereitschaft* am Mammografiescreening nahm nach dem Lesen der Entscheidungshilfe insgesamt leicht ab (von 64 % auf 61 % bei den Frauen < 50 Jahre und von 67 % auf 64 % bei Frauen > 50 Jahre). Ältere Frauen gaben allerdings eher als jüngere Frauen nach dem Lesen an, am Screening teilzunehmen zu wollen. Durch das Lesen der Broschüre änderten insgesamt 20 % der jüngeren Frauen (< 50 Jahre) ihre Teilnahmebereitschaft innerhalb der 3 Kategorien „teilnahmebereit“, „unentschlossen“ und „nicht teilnahmebereit“ (Abb. 2). Bei älteren Frauen (50+) lag der Wert bei 14 % (siehe [1]). Das sind Hinweise, dass die Materialien eine informierte Entscheidung unterstützen.

### Entscheidungshilfen zum Darmkrebsscreening

Es wurden 2 geschlechtsspezifische Entscheidungshilfen (Broschüren) für das deutsche organisierte Darmkrebsscreeningprogramm entwickelt. Folgende Themen werden in den Informationsmaterialien adressiert:

- allgemeine Informationen zum Thema Darmkrebs,
- Informationen zum Erkrankungsrisiko,
- Informationen zum Ablauf der Untersuchungen,
- Aussagen zum Nutzen und möglichen Schaden des Darmkrebsscreenings,
- Informationen zum Vorgehen bei einem auffälligen Ergebnis.

Bei der Darmkrebsfrüherkennung ist die Entscheidung anders gelagert als bei der Brustkrebsfrüherkennung per Mammografie, da die Früherkennung auch das Erkrankungsrisiko senken kann. Zudem stehen Nutzerinnen und Nutzer im deutschen Programm vor 2 Entscheidungen: (a) für oder gegen die Teilnahme und (b) gegebenenfalls für den Stuhltest oder für die Darmspiegelung. In dieser Konstellation wurde bei der Erstellung der Materialien auf ein explizites Instrument zur Präferenzklärung verzichtet und eine einfache tabellarische Zusammenfassung (Option Grid) entwickelt. In dieser sind die wesentlichen Aspekte enthalten, die in der Entscheidungshilfe genannt werden, die Vor- und Nachteile der Optionen „keine Teilnahme an der Früherkennung“, „regelmäßiger Stuhltest“ und „Darmspiegelung“ werden gegenübergestellt [1].

#### Qualitative Nutzertestung.

In der qualitativen Nutzertestung (4 Gruppendiskussionen mit 36 Personen und 8 Experteninterviews) wurde die Broschüre von den Nutzern und Nutzerinnen als sehr informativ bewertet. Manche Nutzerinnen und Nutzer hoben die objektive Darstellung der Vor- und Nachteile der Früherkennung positiv hervor. Einige beschrieben es als verwirrend, dass die Leistungen zur Früherkennung ab einem gewissen Alter eingeschränkt würden, obwohl das Risiko für Darmkrebs mit steigendem Alter zunimmt. Die Broschüre hatte auf manche eine eher abratende als zuratende Wirkung. Der Hauptgrund hierfür war, dass die Nutzerinnen und Nutzer überrascht darüber waren, dass das Risiko, an Darmkrebs zu erkranken, deutlich geringer ist als erwartet und damit auch die Anzahl derer, die von der Früherkennung profitieren könnten. Gleichzeitig würden aber viele die Broschüre wegen der neutralen Darstellung und der Quantifizierung des Nutzens weiterempfehlen.

#### Quantitative Nutzertestung.

An der quantitativen Nutzertestung (Online-Survey) nahmen jeweils rund 1000 Frauen und Männer im Alter von 50 bis 65 Jahren teil, bei denen keine Darmkrebsdiagnose vorlag. Circa 80 % der Männer und Frauen bewerteten die Broschüre insgesamt als gut oder sehr gut. Ein etwas höherer Anteil beurteilte die Broschüre als sehr vertrauenswürdig (fast 90 %) und mehr als 70 % wären bereit die Broschüre weiterzuempfehlen. Nach dem Lesen der Materialien blieb die Bereitschaft, einen Stuhltest oder eine Darmspiegelung durchführen zu lassen, bei Männern und Frauen auf einem ähnlichen Niveau wie vor dem Lesen.

Der Anteil der Befragten, die nicht bereit waren, am Stuhltest teilzunehmen, stieg durch das Lesen von 6 % auf 10 %. Die *Teilnahmebereitschaft* am Stuhltest ging somit um etwa 4 % zurück. Die Bereitschaft zur Darmspiegelung nahm um 2 % zu (vor dem Lesen 54 %, nach dem Lesen 56 %). Innerhalb der Kategorien „teilnahmebereit“, „unentschlossen“ und „nicht teilnahmebereit“ änderten etwa 20 % der Befragten durch das Lesen der Broschüre ihre Intention [2, 18].

### Entscheidungshilfen zum Zervixkarzinomscreening

Da beim Zervixkarzinomscreening je nach Alter unterschiedliche Untersuchungen angeboten werden [3], wurden 2 Entscheidungshilfen (für Frauen im Alter von 20 bis 34 Jahren sowie für Frauen ab 35 Jahren) entwickelt. Die Entscheidungshilfen werden seit 2020 an alle gesetzlich versicherten Frauen der Altersgruppen postalisch versendet. Die Entscheidungshilfen bestehen jeweils aus 20 Seiten mit Abbildungen, Tabellen und Fotos. Beide Broschüren beinhalten Informationen:

- zum Thema Zervixkarzinom allgemein,
- zum Erkrankungsrisiko,
- zum Ablauf der Untersuchungen,
- zur Impfung gegen humane Papillomviren (HPV),
- zu den Vor- und Nachteilen des Zervixkarzinomscreenings in tabellarischer Form,
- zur Bedeutung von Dysplasiebefunden,
- zum Verfahren der Konisation, einschließlich Nebenwirkungen,
- zu den Grenzen der Früherkennung,
- zur Unterstützung der Entscheidung (einschließlich zusammenfassender Gegenüberstellung der Optionen).

Auf ein Präferenzklärungsinstrument wurde hier ebenfalls verzichtet und stattdessen eine rein tabellarische Zusammenfassung der Vor- und Nachteile entwickelt. Sie fasst die wesentlichen Aspekte zusammen, die in der Entscheidungshilfe genannt werden, und stellt die Vor- und Nachteile der Optionen „keine Teilnahme an der Früherkennung“ und „regelmäßige Früherkennung“ gegenüber [3].

#### Qualitative Nutzertestung.

Die* Fokusgruppeninterviews *(4 Gruppendiskussionen mit 26 Frauen) haben ergeben, dass einige Frauen nicht wussten, dass die Früherkennungsuntersuchung auch Nachteile haben kann, wie eine unnötige Konisation (Entnahme eines Gewebekegels aus der Zervix). Zudem war einigen nicht bekannt, dass sich Dysplasien (Zellveränderungen) von selbst wieder zurückbilden können. Ein Teil der Testerinnen fand die Darstellung der Vor- und Nachteile in Tabellen hilfreich, anderen bereiteten die Entwürfe der Tabellen Verständnisschwierigkeiten oder sie zeigten weniger Interesse an Zahlen. Aufgrund dieser Ergebnisse wurden Anpassungen an den Entwürfen vorgenommen.

Obwohl die befragten *Expertinnen und Experten* (*n* = 8) Aufklärung für wichtig erachteten, fand ein Teil die Darstellung der Nachteile zu umfangreich. Sie äußerten die Befürchtung, dass dies Frauen von der Teilnahme am Screening abhalten könne. Zudem wurden Vorbehalte gegenüber dem HPV-Test geäußert.

#### Quantitative Nutzertestung.

An der quantitativen Nutzertestung (Online-Survey) der finalen Entwürfe nahmen rund 2000 Frauen teil. Die Mehrzahl aller befragten Frauen vertraute den Entscheidungshilfen und fand sie verständlich. Die Darstellung der Vor- und Nachteile des Screenings wurde zumeist als ausgewogen empfunden.

4 % bzw. 7 % der Frauen gaben an, dass sie die Darstellung der Nachteile von der Früherkennung abschreckt. Die *Teilnahmebereitschaft* änderte sich nach dem Lesen der Entscheidungshilfe insgesamt aber kaum. Die absolute Teilnahmebereitschaft veränderte sich kaum (vor dem Lesen der Broschüre 67 %, nach dem Lesen 68 %). Es gab relevante Wechsel zwischen den 3 Kategorien. Bei den jüngeren Frauen wechselten ca. 10 % von „teilnahmebereit“ zu „unentschlossen“. In der entgegengesetzten Richtung waren es 8 %. Bei den älteren Frauen sah es ähnlich aus. Insgesamt änderten in allen Altersgruppen rund 23 % der Frauen ihre Intention durch das Lesen der Broschüre. Dies ist auch hier ein Indiz dafür, dass die Materialien die informierte Entscheidung unterstützen.

## Haben die Entscheidungshilfen ihre Ziele erreicht?

Die erstellten Entscheidungshilfen haben zum Ziel, evidenzbasiert, ausgewogen, verständlich und in angemessenem Umfang über das Mammografie‑, Darmkrebs- und Zervixkarzinomscreening zu informieren. Die Materialien sollen eine informierte Entscheidung unterstützen. Dabei werden neben allgemeinen Informationen zum Ablauf des Screenings auch seine Vor- und Nachteile beschrieben. Ebenso galt es in allen 3 Projekten, die Freiwilligkeit an der Teilnahme zu betonen.

Im Rahmen einer ca. 20-seitigen Broschüre konnten nicht alle Informationsbedürfnisse, die bei der Auswertung der qualitativen Studien und Surveys identifiziert wurden, aufbereitet werden. Deshalb finden sich ergänzende und vertiefende Inhalte auf der Website „gesundheitsinformation.de“, auf die in den Broschüren auch verwiesen wird.

### Mammografiescreening.

**Figure 3:**
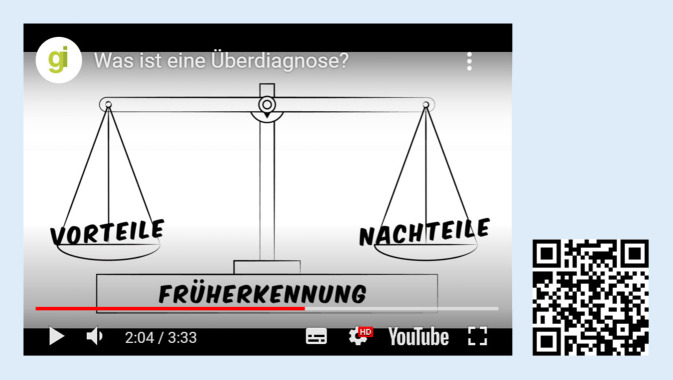

Die Weiterentwicklung des Merkblatts zu einer Entscheidungshilfe wurde von den Fachleuten und den zu befragenden Frauen als gut bewertet. Die Entscheidungshilfe regte Frauen an, sich mit den Vor- und Nachteilen des Mammografiescreenings auseinanderzusetzen. Darüber hinaus wurde deutlich, dass die Entscheidung für oder gegen die Mammografie erfolgen kann. Das Wissen zu Kernaspekten nahm nach dem Lesen der Materialien zu [1, 14, 15]. Die Nutzerinnen schätzten das Thema Überdiagnosen als besonders wichtig ein. Um das Verständnis zu erleichtern, schlugen die Frauen vor, Überdiagnosen anhand eines Beispiels zu erklären. Es wurde allerdings auch angenommen, dass dadurch die Entscheidungshilfe als nicht ausgewogen wahrgenommen werden könnte, da das Thema Überdiagnosen verhältnismäßig viel Platz einnähme. Der Vorschlag der Nutzerinnen wurde aber umgesetzt. Zudem wurde ein Video zur Erläuterung von Überdiagnosen erstellt und auf gesundheitsinformation.de veröffentlicht (Abb. 3). Die Sorge, dass dadurch die Entscheidungshilfe als nicht ausgewogen wahrgenommen werden könnte, bestätigte sich in der abschließenden Testung nicht. Das entwickelte Präferenzklärungsinstrument wurde als Unterstützung der Reflexion gut akzeptiert.

### Darmkrebsscreening.

Die Darmkrebsfrüherkennung gehört zu den wirksamsten Früherkennungsuntersuchungen. Dennoch gibt es in der Bevölkerung unterschiedliche Präferenzen bezüglich der Inanspruchnahme [18]. Das Ziel, neutral über den Ablauf der Darmkrebsfrüherkennung sowie ihre Vor- und Nachteile zu informieren, wird in der vom IQWiG vorgelegten Entscheidungshilfe erreicht. Die vor allem von Fachleuten formulierte Sorge, dass die Darstellung von Vor- und Nachteilen abschreckend wirken könnte, bestätigte sich in der abschließenden quantitativen Nutzertestung nicht. Das entspricht den Ergebnissen einer randomisierten Studie aus Deutschland [20].

### Zervixkarzinomscreening.

Auch beim Zervixkarzinomscreening ergab die abschließende Nutzertestung eine hohe Akzeptanz. Befürchtungen, dass die Früherkennung wegen der Nennung von Nachteilen von den Frauen abgelehnt würde, bestätigten sich nicht. Auf einen großen Teil der Leserinnen wirkten die Entscheidungshilfen sogar eher zuratend. Das Ziel von Entscheidungshilfen ist es, möglichst neutral und ausgewogen zu informieren. Im Laufe des Erstellungsprozesses wurde durchgehend auf diese Ausgewogenheit geachtet. Es ist aber davon auszugehen, dass allein die Konfrontation mit Materialien zum Thema Früherkennung als Empfehlung verstanden wird [3].

## Fazit

Die in den 3 Projekten entwickelten Entscheidungshilfen wurden unter Beachtung der Standards der Risikokommunikation entwickelt: Dazu gehören (a) eine verständliche Beschreibung der Risiken, an einer der Krebsarten zu erkranken (in absoluten Risiken), (b) eine vergleichende Darstellung von Vor- und Nachteilen der Früherkennung (ebenfalls als Vergleich absoluter Risiken) und (c) ein Verzicht auf überredende Elemente und Formulierungen. Die Materialien wurden in den quantitativen Nutzertestungen weitgehend übereinstimmend als gut verständlich, informativ und hilfreich für die Entscheidungsfindung beurteilt.

Die Materialien führten bei 10–20 % der Befragten vor und nach dem Lesen zu einer (geäußerten) Änderung der Intention zur Teilnahme. Diese Ergebnisse geben einen Hinweis darauf, dass eine informierte Entscheidung unterstützt wurde. Die durchschnittliche Teilnahmebereitschaft blieb weitgehend unverändert.

Das IQWiG hatte in seinen Abschlussberichten zu den Materialien eine regelmäßige Evaluation und Weiterentwicklung empfohlen. Im Herbst 2021 hat der G‑BA das IQWiG mit einer Evaluation der Materialien zum Darmkrebsscreening beauftragt [21]. Die Informationsmaterialien sollen insbesondere in Hinblick auf Akzeptanz, Verständlichkeit für unterschiedliche Personengruppen, Einfluss auf das Inanspruchnahmeverhalten sowie die ärztliche Nutzung im Beratungsgespräch untersucht werden. Neben Inhalt und Umfang sollen dabei auch alternative Formate und Verteilungskanäle evaluiert werden. Die Ergebnisse werden in eine Überarbeitung einfließen.
